# Visibility graph based temporal community detection with applications in biological time series

**DOI:** 10.1038/s41598-021-84838-x

**Published:** 2021-03-11

**Authors:** Minzhang Zheng, Sergii Domanskyi, Carlo Piermarocchi, George I. Mias

**Affiliations:** 1 Biochemistry and Molecular Biology, Michigan State University, East Lansing, MI 48824 USA; 2 Institute for Quantitative Health Science and Engineering, Michigan State University, East Lansing, MI 48824 USA; 3 Physics and Astronomy, Michigan State University, East Lansing, MI 48824 USA

**Keywords:** Systems biology, Time series, Computational biology and bioinformatics

## Abstract

Temporal behavior is an essential aspect of all biological systems. Time series have been previously represented as networks. Such representations must address two fundamental problems on how to: (1) Create appropriate networks to reflect the characteristics of biological time series. (2) Detect characteristic dynamic patterns or events as network temporal communities. General community detection methods use metrics comparing the connectivity within a community to random models, or are based on the betweenness centrality of edges or nodes. However, such methods were not designed for network representations of time series. We introduce a visibility-graph-based method to build networks from time series and detect temporal communities within these networks. To characterize unevenly sampled time series (typical of biological experiments), and simultaneously capture events associated to peaks and troughs, we introduce the Weighted Dual-Perspective Visibility Graph (WDPVG). To detect temporal communities in individual signals, we first find the shortest path of the network between start and end nodes, identifying high intensity nodes as the main stem of our community detection algorithm that act as hubs for each community. Then, we aggregate nodes outside the shortest path to the closest nodes found on the main stem based on the closest path length, thereby assigning every node to a temporal community based on proximity to the stem nodes/hubs. We demonstrate the validity and effectiveness of our method through simulation and biological applications.

## Introduction

Longitudinal behavior is an inherent aspect of all biological systems, and has been widely investigated in various contexts, such as systems biology^1^, metabolic pathway analysis^2^, and, recently, gene expression^3^. With the development of novel technologies in sequencing, mass spectrometry and other omics, multi-level biological time series are becoming easier to obtain. An important example is provided by longitudinal data from personal health monitoring devices. Recent studies have shown that omics time series have a wide range of applications in personal health and precision medicine. Multi-omics time series data can be used in precision health^4^, and have provided insights into the onset of type 2 diabetes mellitus^5^ and lung development^6^. Omics time series can also be used to monitor health events, changes in physiological states^7,8^ and in molecular and medical phenotypes^9^. The rapidly increasing availability of biological time series requires new methods to integrate different types of data, analyze them, and interpret the results in a fast and informative way. Many platforms for multi-biological and multi-omics data integration have been developed, including software such as DAVID^10^, Galaxy^11^ and GenePattern^12^, our recent frameworks MathIOmica^13^ and PyIOmica^14^, which incorporate time-series categorization, and many more.

While typical time series analysis utilizes linear models, non-linear topological methods can provide additional insights into complex temporal behavior. Multiple recent efforts have used networks and graph theory to analyze time series. Network analysis offers a multi-level perspective that can capture non-linear behavior, identify motifs, quantify non-periodic recurrence, and represent the dynamics at different scales^15–22^. Time series are transformed into networks that conserve their topology in the presence of noise and identify noise-independent temporal structures. The network characteristics reflect the equivalent time series temporal structure, including non-linearity and chaotic behavior, and can be used as features to identify trends and build machine learning models, and potentially allow for more accurate learning approaches^23,24^.

Currently, several methods exist for transforming time series to complex networks. For example, complex networks have been constructed from pseudo-periodic time series by Zhang and Small^15^, who used single nodes to represent cycles, and introduced a correlation-based threshold to link node pairs. Another effective and efficient approach is to consider time series points as a series of sequential intensity bars that are then connected based their inter-visibility to obtain a *visibility graph* (VG) representation of the time series^16^, which recently attracted great interdisciplinary interest^17,25^.VGs have been used in diverse time series studies, including investigations into natural phenomena such as hurricanes^26^ and earthquakes^27–29^, finance^22,30^, solar wind^31^ and solar activity^32^, as well as for physiological signal pattern analyses, such as heartbeat^33^, electroencephalograms (EEGs)^25,34,35^, epilepsy^36–38^, and fMRIs^39^.

There are two types of VG typically considered: (1) the Natural Visibility Graph (NVG) and, (2) the Horizontal Visibility Graph (HVG)^40^. To construct a VG, we consider $\{s \left( t_x \right) ; t_x=1,2,3,\dots ,N\}$ as an *N* time point series in temporal ordering. The VG is obtained by first representing the time series points as *N* nodes in a network, where nodes *i* and *j* represent times $t_i$ and $t_j$, with intensities $s(t_i)$ and $s(t_j)$ respectively. Edges are constructed by joining nodes *i*, *j* if any other intermediate time point $t_k$, such as $t_i< t_k < t_j$, has intensity $s(t_k)$ that satisfies the following conditions for NVG and HVG respectively:

1 $$\begin{aligned} \begin{aligned} \text{ s }(t_k) < s(t_j)+(s(t_i)-s(t_j))\frac{t_j - t_k}{t_j - t_i} \qquad \text {NVG,}\\ \text{ s }(t_i), s(t_j) > s(t_k) \qquad \text {HVG.} \end{aligned} \end{aligned}$$

Here, in the NVG formulation, an edge is added connecting nodes *i* and *j* if any other time point $t_k$, between $t_i$ and $t_j$, has a corresponding intensity $s(t_k)$ that lies below the line connecting $s(t_i)$ and $s(t_j)$ (i.e. there is a direct line-of-sight between these peaks). The HVG has a simpler edge construction condition: an edge is added connecting nodes *i*, *j* only if all intermediate intensities $s(t_k)$ are less than both $s(t_i)$ and $s(t_j)$.

NVGs and HVGs are connected networks. The VG conserves the structure of the time series in the graph topology^16^. However, the HVG original constructions do not account explicitly for the potential effects of uneven time sampling or missing time points. In the NVG such uneven sampling results in different visibility of timepoints (changes in viewing angles in the construction), which implicitly incorporates the effect of time distances, but without explicitly weighting the edges the actual distance between nodes cannot be accounted for. In realistic situations outside a laboratory setting, uneven sampling occurs often. This may be due to technical limitations (for example in mass spectrometry proteomics technical replicates may still sample different proteins and lead to missing data and hence uneven sampling), or limitations in subject participation (for example in clinical trials and human subject research, the subjects’ work-dependent schedules may affect their regular participation). These shortcomings limit the traditional VG application in biological/medical time series analysis. Another limitation of the traditional VG is the inability to simultaneously capture peaks and troughs (points below the baseline). For example, the VG maps sinusoidal and cosinusoidal time series to different graphs, but these two kinds of time series should be considered equivalent up to a change of phase.

Another challenge in network representations of biological time series is the lack of specific methods for detecting temporal communities in individual signals. A community is defined as a group of nodes, where the nodes within a community are tightly connected, whereas the nodes between different communities have loose connections^41^. Each community in complex networks representing a biological time series thus identifies nodes with similar temporal behavior that are likely to represent the same underlying biological system state. Thus, these temporal communities correspond to sets of timepoints within a single biological signal that show consistent temporal behavior, corresponding to a distinct biological state. As we move from one temporal community to the adjacent next community the signal characteristics change and we effectively transition into different biological behavior or state. One highly effective approach for identifying communities is to compare the actual number of intra-community edges to what one would expect by a random placement of the edges^41–43^. This approach is based on the assumption of a random graph null model. However, VGs cannot be considered as random graphs, even if a VG is constructed based on a random time series. This is due to the sequential nature of the nodes, the resulting connected graph, and the underlying degree distribution^40^.

In this investigation, we introduce the method of “weighted dual perspective visibility graph” (WDPVG) for mapping time series to complex networks. Our WDPVG approach considers uneven sampling effects, and simultaneously captures peaks and troughs of time series. Previously, VG edge weights had been assigned based on the arctangent of ($(s_j-s_i)/(t_j-t_i)$), which computes the “view angle” along the direct line-of-sight connecting one intensity peak to another^36^. Our method provides multiple choices for the edge weights: (1) the Euclidean distance between nodes/intensity peaks, (2) the tangent of the view angle between two nodes, (3) the time difference between time points corresponding to connected nodes, or (4) none. We then combine the natural view perspective VG with the “reflected view perspective VG” introduced in the methods below to create a complex network that can capture both the positive and negative intensities changes. We note, that this is the first time that Euclidean distance has been used for edge weights in VGs, to the best of our knowledge.

We also provide a new automated VG community detection method, which is based on shortest path calculations between VG nodes. Our method is suitable for VGs as it does not depend on random graph null models, which are commonly used in other approaches such as Newman’s method^43^. Briefly, as described below, we compute the shortest path of the VG between start and end nodes as a main stem. The nodes on the main stem are seeds for communities, and we then aggregate nodes outside the shortest path to their most proximal seed nodes on the main stem, where proximity is determined using graph path lengths. In utilizing the shortest path as seed nodes, we are using the time points that display the peaks of highest intensity. These peaks are the dominant features in the signal, and represent prominent temporal behaviour. Biologically the nodes in the shortest VG path correspond to drivers of the signal’s response.

We used various simulated time series to test our method effectiveness, and demonstrated that our automated method has high tolerance for uneven sampling and signal noise. Our comparison of our method to traditional community detection methods, such as Girvan–Newman^41^ and Louvain^44^, indicated that our approach is more suitable for VGs. To show that our method can capture biological processes we also applied it to several experimentally obtained time series, longitudinal multi-omics data from blood components in prediabetics (cytokines, glucose and haemoglobin A1c)^5^, saliva omics data (mean gene expression)^14^ and signals from wearable biosensors (radiation exposure)^45^.

The methods of building WDPVG and visibility graph based community detection are available as a module of the open source Python package PyIOmica^14^. The dataset and codes we used as described below are available with a Python notebook, publicly available online at https://doi.org/10.5281/zenodo.3693984.

In summary, in this study we: (1) Provide, to the best of our knowledge, the first dedicated method to calculate communities in visibility graphs, (2) define the WDPVG visibility graph construction that allows for an equal treatment of events above or below a baseline, (3) utilize our method to identify changepoints (i.e. community boundaries) in biological signals corresponding to perturbation-induced changes.

## Materials and methods

### Weighted dual perspective visibility graph (WDPVG)

The VG can characterize time series in terms of complex network theory as it can inherit the structure properties of the time series data from which it was created. VGs are robust to noise and not affected by the selection of method parameters (e.g. cutoffs/thresholds)^46^. VGs have been widely applied in many fields^22,36,47^. However, as we discussed above, the VG has two disadvantages: first, it does not consider the effect of uneven sampling; second, it cannot capture the time series changes below a zero baseline. Here we provide a new method, WDPVG, to overcome these limitations that restrict VG applications in biological time series analysis.

We use the following four steps to create WDPVGs, utilizing auxiliary natural visibility graphs (NVGs).

*Step 1: NVG construction.* We create an NVG from time series $\{s \left( t_x \right) ; t_x=1,2,3,\dots , N\}$ as it was described above by Eq. (1), using the NVG mapping criteria.

*Step 2: Assign edge weights between two nodes.* In our software implementation we provide flexible choices for the edge weight between two nodes, including no weighting (NO), Euclidean distance (ED, Eq. 2), the tangent of the view angle (TAN, Eq. 3), or the time difference (TD, Eq. 4):

2 $$\begin{aligned} w_{ij}= & {} \sqrt{(s(t_i) - s(t_j))^2 + (t_i - t_j)^2} , \end{aligned}$$

3 $$\begin{aligned} w_{ij}= & {} \left| \frac{s(t_i) - s(t_j)}{t_i - t_j}\right| + 10^{-8}, \end{aligned}$$

4 $$\begin{aligned} w_{ij}= & {} |t_i-t_j|, \end{aligned}$$

where, $w_{ij}$ represents the weight of the edge between nodes *i* and *j*, which correspond time points $t_i$ and $t_j$ respectively, in time series $\{s \left( t_x \right) \}$. In Eq. (3), we added the offset $10^{-8}$ to account for the case $s(t_i) - s(t_j) = 0$. The algorithm implementation details are available in the documentation of the functions in PyIOmica. In this manuscript, we use Euclidean distance between nodes as the edge weight. The choice of distance depends on the application. For example, if considering sequential timepoints as a series of states, where time differences have secondary importance, then weights for the edges could be ignored. In contrast, the Euclidean distance takes explicitly into account both intensity differences and time differences, compared to other distance options, and hence may offer an advantage in uneven sampling cases, and in anomaly detection applications where the intensity differences should be taken into consideration.

After Step 2, We compute the adjacency matrix, *A*, of the normal perspective NVG.

*Step 3. Compute the reflected perspective NVG.* We invert the time series $\{s \left( t_x \right) \}$, by reflecting across the time axis, i.e. for each $s(t_i)$ in $\{s \left( t_x \right) \}$, let $s^{\prime } (t_i) = -s(t_i)$, where we obtain the inverted time series $\{s^{\prime } \left( t_x \right) \}$. We then repeat Steps 1 and 2 for $s^\prime (t_x)$ to get the reflected perspective NVG and the adjacency matrix $A^{\prime }$.

*Step 4. Combine the normal perspective NVG and reflected perspective NVG.* For any pair of *i*, *j*, elements $A_{ij}$ and $A^{\prime }_{ij}$ have two possible relationships: either $A_{ij} = A^{\prime }_{ij}$, or one of them is 0 but the other one is non-zero. We can combine the *A* and $A^{\prime }$ to get the WDPVG adjacency matrix $A^{d}$ by the following criteria:

5 $$\begin{aligned} A^{d}_{ij} = \max { \{ A_{ij},A^{\prime }_{ij} \}} \end{aligned}$$

If we use the HVG mapping criteria, i.e. $\text{ s }(t_i), s(t_j) > s(t_k) \qquad t_i< t_k < t_j$, instead of the NVG mapping criteria, we can obtain the weighted dual perspective horizontal visibility graph.

It is important to note that in case that either we are not interested in changes below the baseline, or that the intensities of the time series are all non-negative, the normal perspective weighted VG is enough, and we do not need to create a WDPVG.

### Shortest path based community detection

The central problem solved in this section may be summarized as: Given a time series $s(t_x)$, and assuming a visibility graph representation *g*,(where *g* is constructed as an NVG, or HVG, or WDPVG), segment *g* into *k* segments (communities), where $1\le k \le N)$, to minimize the shortest path length of nodes assigned to each segment *k*, to nodes on the shortest path in *g*. The shortest path in a VG between the start node (corresponding to first time point) and end node (corresponding to last time point) identifies a bundle of nodes which have high intensities, and thus is the determining factor for the entire network structure. This shortest path acts as a stem for community identification in VG. Our method chooses the shortest path between start node and end node in VG as the main stem, and each node on this stem is a natural hub of a community, as described below.

*Step 1. Construct the shortest path between VG first and last nodes.* Given a VG *g* with *N* nodes, construct the shortest path $\{{v}^{s}_i; i = 1,2,3,\dots ,k \}$ (comprised of *k* nodes) between the start node and end node in *g*.

*Step 2. Hub construction.* We define the nodes outside the shortest path as $\{ v^{o}_j; j = 1,2,3...m\}$, where $m=N-k$. For any node $v^{o}_p$ in $\{ v^{o}_j \}$, we compare the shortest path length between $v^{o}_p$ and each node in $\{ v^{s}_i \}$, to identify the minimal path length value $l_{pq}$ and corresponding node $v^{s}_q$ in $\{ v^{s}_i \}$. $v^{o}_p$ is then assigned to the community whose hub is $v^{s}_q$. If there are more than one hubs corresponding to the minimal value, we always choose the “left” hub, which corresponds to the earlier time point, as the target community’s hub. We then iterate through all nodes in $\{ v^{o}_j \}$, to get the community structure.

*Step 3 (optional). Hub merging*. Finally, we measure the shortest path length between any pairs of hubs, i.e. the nodes in $\{ v^{s}_i \}$, and if the shortest path length between them is less than a chosen cutoff, $\epsilon$, we then combine the two associated communities to obtain the final community structure. Normally, the minimal $\epsilon$ is the cutoff for which the network has the same number of communities as the number of hubs. Similarly, the maximal value of $\epsilon$ corresponds to the case where the whole network becomes a single community. By changing the value of $\epsilon$ between minimum and maximum, one can modify the community structure, i.e. the number of communities. In our Python implementation, we have provided the following options for cutoff selection: (1) with or without cutoff, (2) fixed cutoff, or (3) automatically selected cutoff. The cutoff is selected as the largest $\epsilon$ for which the distance between hub nodes is smaller than the median shortest path length distance within the VG. The merging feature is unique to our method.

An additional feature of our method is that we can choose the direction of how the nodes are connected in the community construction. Specifically, we can restrict the node $v^{o}_p$ in $\{ v^{o}_j \}$ to only link to the community with hub $v^{s}_q$ for which the corresponding time point $t_q$ is earlier than the time point $t_p$ corresponding to $v^{o}_p$. This feature essentially imposes a causality condition, where time points only depend on other past time points, and not future ones. It is important to keep time order in the community detection for characterizing biological time series from living systems. We have allowed flexibility in the implementation of the method, so the user can also choose node linking directions as earlier side, later side or both sides - this may be required for systems where there is time reversal symmetry.

**Figure 1:**
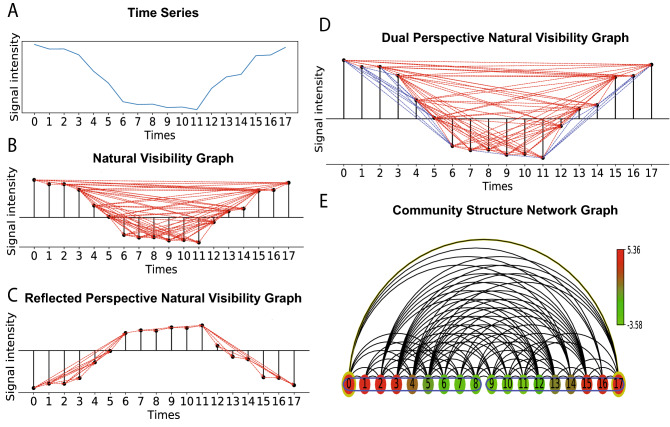
Illustration of the construction of weighted perspective visibility graph and community structure based on the shortest path length community detection method. (**A**) is the simulated time series cosine signal with 40% noise in intensity. We construct the weighted NVG and reflected NVG, where the edge weights are based on Euclidean distance. Edge connections of NVG and reflected NVG are illustrated in (**B**) and (**C**) respectively. The dual weighted perspective visibility graph is created by combining NVG and reflected NVG, as shown in (**D**), where the links with red color come from NVG, and the blue links come from the reflected NVG. Using our shortest path length based community detection method, we can find the community structure of the time series, as shown in (**E**). The time points in the same community are encircled by a blue outline, and the color of the nodes represents the signal intensity. The time series separates into two communities that capture the cosine signal’s periodicity.

Figure 1 provides a simple illustration of how our method works. A simulated time series is created from a cosine wave signal mixed with 40% random noise (Fig. 1A). Then, we construct the weighted NVG and the reflected perspective NVG in Fig. 1B,C. Afterwards, the weighted dual natural visibility graph is created by combining the NVG and the reflected perspective NVG (Fig. 1D). We use our community detection method to the graph in Fig. 1E showing the communities corresponding to the original time series.

### Simulation

We used simulated time series to evaluate our method. We illustrate here three types of time series: cosine, square, and saw-tooth wave signals. Then we added different intensity random noise to each of these signals to test the tolerance of the community detection to noise. We also randomly removed different percentages of time points from these time series to check the robustness to missing data and the resulting uneven sampling. We then built the WDPVG for these time series, and detected communities using our method. The results of our method were compared to traditional community detection methods such as the Louvain method, which is a widely used method of fast greedy optimization of modularity^44^, and the Girvan–Newman hierarchical method which is based on centrality notions^41^. The Louvain method was implemented using the Python python-louvain package, (https://github.com/taynaud/python-louvain). The Girvan–Newman method is available in NetworkX, which is the most popular open source network analysis package in Python^48^. Finally, we compared the community structure obtained by our algorithm under the different types of edge weights, as well as the algorithm’s performance for different amplitudes and frequencies.

Our community structure detection algorithm includes two parts. The first part of the algorithm finds the shortest path length. The time complexity of this part is $\mathcal {O}(\left| E\right| + N\log {}N)$^49^, where $\left| E \right|$ is the number of edges and *N* is the number of nodes in the VG, *g*. The second part assigns *m* nodes outside the shortest path to the *k* nodes on the shortest path between the start and end nodes. The time complexity of this part is $\mathcal {O}(km)$, which equals $\mathcal {O}((N-k)k)$ . Hence, the total time complexity of our algorithm is the sum of these two parts, $\mathcal {O}(\left| E\right| + N\log N + (N-k)k))$.

### Experimental biological time-series applications

We also compared the results of our method to the Louvain and Girvan–Newman methods when applied to several experimentally acquired biological datasets. These datasets used are summarized below.

*Saliva Set (DS1)* We used a saliva RNA-sequencing dataset we generated, that was obtained from a clinical trial monitoring individualized response to the standard 23-valent pneumococcal polysaccharide vaccine (PPSV23)^50^. The saliva was sampled from a healthy individual. We had first carried out a 24-h hourly sampling to establish a normal physiological state baseline. Then, we repeated with another 24-h hourly sampling that also included vaccination with pneumococcal vaccine (PPSV23) to assess response to the vaccine. The vaccine was administered approximately 3.5 h following the first hourly sample. Approximately 7.5 h after vaccination, the individual reported having a fever that lasted about 4 h. Here, we analyzed the differences between the two 24 h periods: (1) the first 24 h hourly sampling (Sal$_1(t)$) and (2) the 24 h hourly sampling that included vaccination (Sal$_2(t)$). We then constructed the paired difference time series $\Delta$, where for each timepoint *i* for each gene $\alpha$, $\Delta _{\alpha \text {Sal}}(t_i) = \text {Sal}_{2\alpha }(t_i)-\text {Sal}_{1\alpha }(t_i)$. We carried out a categorization into groups and subgroups of gene expression based on these data (see online Python notebook, and previous discussion using PyIOmica^13,14,51^). For a given subgroup of genes, we constructed the mean time series across the members of this subgroup. We then built the WDPVG and compared the different temporal community detection methods results on this time series (see also online Python notebook for code and data at https://doi.org/10.5281/zenodo.4542567).

*Diabetes Set (DS2)* The second dataset came from personal multi-omics profiling data (e.g. including blood measurements of A1C, fasting glucose and selected immune cytokines etc.) from individuals with Type 2 diabetes mellitus at its earliest stage^5^. As an example, we chose one individual’s A1C, fasting glucose and selected immune cytokines data from the rich dataset, as reported by the authors. These time series include 14 time points with different healthy condition. We constructed the VG from each of the time series and detected its corresponding communities, to assess whether our method can capture the physiological status of the subject for each of these time series.

*Radiation Exposure Set (DS3)* Finally, we analyzed a radiation exposure time series dataset from wearable biosensors^45^. The data collected were hourly personal radiation exposure, assessed by a wearable biosensor for more than 100 days. We chose one day spans (24 h, from 12 am to 11 pm) as the natural time window. We then analyzed separately four days when the individual of this study had flight activity, and the radiation reported on these days was higher than non-flight days. We then applied our methods to assess if we can detect the radiation events as community structures.

**Figure 2:**
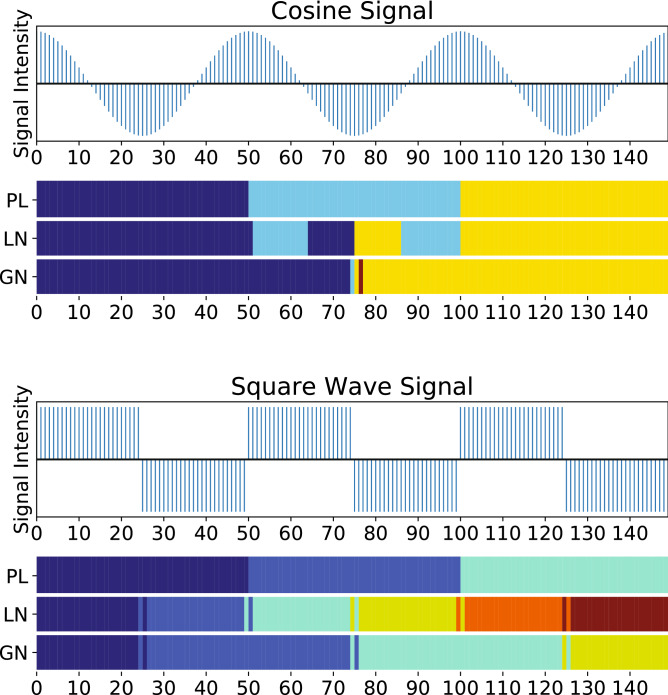
The intensity and communities of cosine (top) and square wave (bottom) signal time series. The communities obtained by our method (PL), Louvain method (LN) and Girvan–Newman method (GN) are represented with color bars, with time points in the same community having the same color. The communities obtained with our automated method on both cosine and square wave time series are capturing the signal periods. The Louvain method captures the characteristics of the square wave signal but shows temporal timepoint mixing in communities on the cosine signal. The Girvan–Newman method captures the periods of the two time series with some errors at the boundaries.

## Results

### Simulation

To investigate whether our method captures periodic features we simulated well defined periodic time series. We compared our path-length based method (PL) with two widely used community detection method, the Louvain method (LN)^44^ and the Girvan–Newman method (GN)^41^. Figure 2 shows the signal intensity and communities for the cosine and square wave signals’ time series (top and bottom, respectively). The communities our method detected matched exactly with the signal periods. The Louvain method also captured precise periodic features in the square wave signal, where it assigned communities corresponding to half periods. However, the Louvain method obtained some unmatched results in the case of the cosine signal time series. Finally, the Girvan–Newman method obtained coarser results compared to the other two methods.

**Figure 3:**
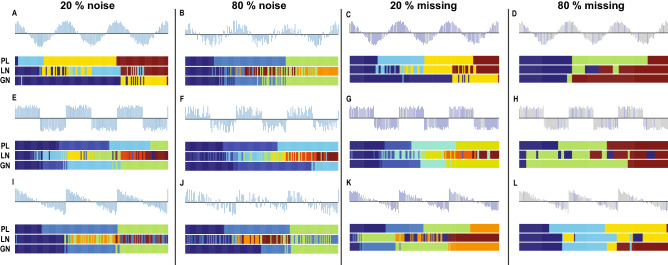
Community structure of different simulated signal time series. We simulated 3 types of signals: cosine time series (**A–D**), square wave time series (**E–H**) and saw-tooth wave time series (**I–L**). The community structure is represented by color bar, with time points in the same community assigned the same color. For each kind of signal, we also added 20% and 80% noise (the first and second subplots of each row respectively) to test the tolerance to noise. We also randomly removed 20% and 80% time points (the third and fourth subplots of each row respectively, where grey bars in the signal plots represent the removed time points, in the case of having 20% noise in the data) to check the robustness to missing data and uneven sampling. We compared our method (PL, first color bar of each subplot) with the two traditional community detection methods, Louvain method (LN, second color bar) and Girvan–Newman method (GN, third color bar). Our method identifies community structures matching with the characteristics of the various time series, even in the presence of noise or missing data.

In addition, Fig. 3 shows results on the tolerance to noise and missing data for cosine wave (Fig. 3A–D), square wave (Fig. 3E–H), and saw-tooth wave signals (Fig. 3I–L). Compared to the Louvain and Girvan–Newman methods, our method displays higher tolerance in either situation of noisy signals or uneven sampling. Whenever we added noise from 20 to 80%, or additionally removed time points from 20 to 80% (in the case of having 20% noise in the data), our method still captured the periodic changes. However, we do notice a change in the number of communities. In particular, adding noise may lead to merging of adjacent communities, for example merging the communities corresponding to different periods in the signal. To the contrary, the results of the two traditional methods were irregular, with coarsely defined communities and multiple nodes in communities unmatched with the corresponding signal’s period. Even though the Louvain method worked well in the perfect square wave signal time series (e.g. without noisy and uneven sampling), the method showed low tolerance to noise or time point removal. Compared to our method that may merge communities, the Louvain method also appears to add additional spurious communities (or multiply-segmented communities), and displays high sensitivity to the noise structure.

**Figure 4:**
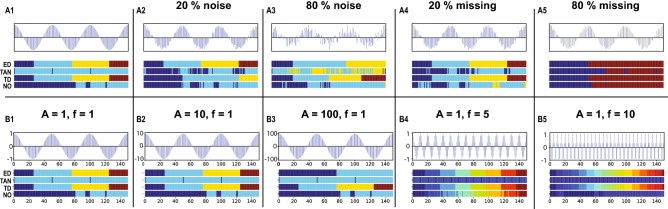
Community structure for different edge weights, amplitudes and frequencies. We compared the path length based algorithm (PL), using a weighted dual perspective visibility graph (WDPVG), for 4 types of edge weights: Euclidean distance (ED), the tangent of the view angle (TAN), the time difference (TD) and no weighting (NO). We simulated sinusoidal signal time series with different noise levels and missing data (20% and 80%), and obtained the communities using the PL algorithm (**A1–5**). We also changed the amplitudes, A from 1 to 100 (arbitrary units), and the frequencies, f, from 1 to 10, and detected the communities of the WDPVG from the signals (**B1–5**). The community structure is represented by a color bar, with time points in same community assigned the same color. The edge weights of ED and TD work well among the 4 types edge weights, and the PL algorithm shows robustness under amplitude or frequency changes.

We compared our path length based method for the different types of edge weights (ED, TAN and TD, see Eqs. 2, 3 and 4 respectively), as well as the no weighting method (NO, e.g. setting all edge weights to 1). The community detection results from building the WDPVG for cosine signals with different amount of noise or missing data under different types of edge weights are shown in Fig. 4A1–5. Our algorithm works well with edge weights of Euclidean distance and Time difference. The community structure for TAN and NO edge weights do not represent effectively the corresponding signal’s period. These methods result in non-contiguous communities, mixing different signal regimes. The results from changing the amplitudes of the signals from 1 to 100 (arbitrary units), and multiplying the signals’ frequencies by 5 and 10 are shown in Fig. 4B1–5. Using ED and TD edge weights still provides community structure corresponding to periodic signals, indicative of robustness of the algorithm under amplitude and frequency changes. The community structure does not change for small amplitude modifications, but shows modifications for the ED weights for larger amplitudes (merging of adjacent communities), while the TD method results are not affected—effectively removing the time change influence with dominant amplitudes receiving high weights in the ED method. The different frequency modifications change the number of communities, corresponding to the periodicity changes, with the TD and ED methods both matching the signals’ periods for different frequencies. The TD method is robust to changes in amplitude as it is independent of amplitude.

**Figure 5:**
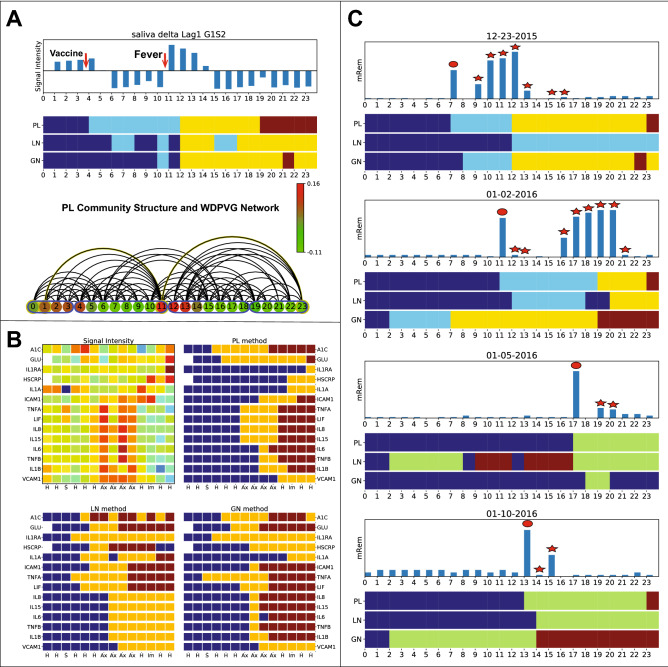
Results of our visibility graph based community detection method applied to experimental biological datasets. (**A**) Shows the results of applying different methods to an individual’s saliva omics time series before and after their vaccination, over a 24 h period. The time series indicates average gene expression for a subset of genes with autocorrelated behavior. The VG nodes’ color in (**A**) bottom represents the intensities of the time series, and nodes belonging to the same detected community are framed by a blue border. The communities correspond well to the four physiological subject states of biological significance: prior to vaccination, after vaccination, a post vaccination fever period and resolution, post fever relative recovery. (**B**) The standardized intensities of A1C, fasting glucose and selected immune cytokines time series from a subject with Type 2 diabetes (**B** top left) and the community structures computed individually for each signal are shown for different methods. The data are ordered temporally from left to right, and physiological states are indicated per timepoint as one of H (healthy), S (stress), Ax (antibiotic regiment) and Im (immunization) states. For the experimental data, warm colors indicate higher and colder colors lower intensities respectively. In the methods’ results, nodes belonging to the same community are depicted with the same color bar. The communities structure reflects the changes in physiological state that result from molecular intensity differences. (**C**) The radiation intensity data from wearable biosensors in four separate days including flight activity are shown. The red disc indicates that the radiation was recorded during the airport carry-on luggage check. Stars represent radiation monitored during flight timepoints. Again, nodes in the same community are indicated with the same color in the horizontal bars. The PL community structures during each of these four days indicate the periods of varying radiation exposure, and correctly identify the onset of the exposure.

### Experimental biological time-series applications

We then applied our method to the experimentally acquired biological time series summarized in the method section above. First we used our method to detect communities from the saliva omics monitoring experiment, DS1. We chose genes signals from the data displaying autocorrelation at lag 1 (simulation adjusted *p* value< 0.01), and calculated the average across the signals for each time point. The average signal intensity is shown in Fig. 5A top. We then built the WDPVG from this signal and we obtained temporal communities using the different methods in Fig. 5A bottom. The community structure was found to reflect the four physiological states over the 24 h: (1) pre-vaccination baseline, (2) post-vaccination to fever onset, (3) fever onset to resolution, (4) post vaccination baseline. The PL method showed alignment of the changing physiological state of the subject with the communities detected. While the LN and GN methods also performed well, they displayed, however, some mixing of timepoints across physiological states.

The analysis of the Type 2 diabetes dataset, DS2, focused on results previously reported (see Fig. 6B of the manuscript by Zhou et al.^5^). The data included signals of A1C, fasting glucose and selected immune cytokines. There are no negative values in the dataset, so we built a normal perspective NVGs from each entry, and obtained its corresponding community structure. Figure 5B top left shows the heatmap of standardized reported intensities (i.e. the results of Zhou et al.^5^, with the communities structures heatmaps, shown for PL, LN and GN methods as well in Fig. 5B respectively. The community structures detected for DS2 reflect the change of time series intensities changes. We note that our community detection method does not require standardization of the raw data. The community changes capture the status changes in each signal, while effectively filtering out the noise in the data. The PL method results overall reflect the respective durations of the different physiological states (healthy, stress and antibiotic regiments). Qualitatively, the LN and GN methods also yied separations, but with mixing across physiological states.

Finally, we applied our method to four separately days’ radiation exposure measurements from DS3. We show the four separate day results with high radiation exposure changes (when the individual was traveling by flight on these days) in Fig. 5C. The community structures of these four days all capture the radiation exposure changes, acting as an adverse event detector. Again, the PL method is more concordant with the radiation exposure timeframes overall, without mixing of timepoint in the communities.

## Conclusion

We have introduced new methods to characterize graphs derived from time series through the application of VGs. We have introduced WDPVGs that combine normal perspective and reflected perspective visibility graphs, so that the peaks and troughs of a time series can be simultaneously represented. The WDPVGs also take into account uneven sampling effects through weight assignments to the edges. The WDPVG approach thus produces a graph that captures well the characteristics of the underlying time series.

We have also developed a new method to detect communities of nodes in a VG. Our VG community detection method is based on the graph's geometry and considers the shortest path from the start node to the end node. The method does not assume a random graph null model. This makes the method advantageous and more appropriate for all kinds of VGs (e.g. NVG, HVG or WDPVG), because VGs cannot be compared to random graphs due to the sequential nature of time points.

The several simulated time series we used to test our WDPVG and VG community detection methods supported their validity. Our methods also showed high tolerance for uneven sampling and signal noise. Our PL community detection method showed robustness to noise compared to traditional community detection methods such as the Louvain and Girvan–Newman methods. Overall, the results suggest that our approach is well-suited for community detection within VGs.

The application of our automated method to experimental biological datasets gave examples of how the method may be used to identify temporal communities that correspond to biological states (e.g. physiological state of a subject, changes in molecular measurements due to vaccination, or detection of radiation exposure). The method has great potential not only for detecting the boundaries of biological temporal states, but for medical implementation in detecting potential adverse medical events from temporal measurements.

The methods presented are used to identify communities in individual signals, and the community boundaries can be considered as changepoints between different states (e.g. healthy biological signal versus a disease response signal behavior). The changepoints may correspond to anomalies, and the individual communities to motifs. In future work, we plan to extend these methods to compare community structure across different signals and define distance measures that will allow for graph-based classification of multiple signals. Furthermore, we plan to explore additional refinements, including rescaling of intensity and time based on the sampling rate to optimize community detection for weighted VGs. Another extension is to investigate the effect of missing peak data in the community detection. The missing data simulation results (Fig. 3C,D,G,H,K,L) show that in most cases our approach is not sensitive to missing peak values which are in general the community hubs. In the case that missing data correspond to pulse-like single peaks/spikes, with no additional features in the signal, then the particular signal spike characteristic cannot be detected. In the special case where missing peak values are much larger than other values, the community structure may also be changed. A sliding time window approach providing smoothing may address such issues with missing data corresponding to signal peaks and will be further investigated. Additionally, we plan to further automate the optimization of node-merging on the main hubs when constructing the communities, to obtain the most stable set of communities (the current selection is made with either a fixed cutoff, or with a cutoff constrained by the median shortest path distance).

There has been extensive interest in investigating the geometric aspects of community detection in graphs. Notably, in recent work Sia, et al. have proposed a novel geometric approach that is based on evaluating Ollivier-Ricci curvature on graphs to obtain robust network communities^52^. We plan to further extend our work to investigate applying geometric methods to VGs, for example, considering the Ollivier-Ricci curvature of the community hubs in our VG algorithm to optimize hub merging, identifying changepoints, and also building VG community hierarchies. Such hierarchies may be used in clustering biological time series with communities that display different temporal characteristics across different time scales, and identifying common patterns across multiple signals.

We have implemented a graph-based method to explore the community detection for graphs representing time series, building on previously established visibility graph approaches. There are also many other algorithms utilizing analytic methods for similar tasks, for example segmentation algorithms^53,54^. A notable recent implementation is the FLOSS (Fast Low-cost Online Semantic Segmentation) algorithm, which also is visually similar to our approach, using ‘Arcs’ as connectivity variables, in contrast with our method using visibility connections^54^. Since we have taken a graph-based approach, we use graph terminology: for example we refer to ‘temporal community detection’, instead of ‘regime discovery’ or ‘semantic segmentation’ which may be considered as analogous constructions, though with rather different underlying methodologies. Our algorithm offers an extension to time series graph-based methods, further extending the multiple methods available for time series analysis, providing different perspectives that can be particularly beneficial to the complexities of biological time series analysis.

## Data Availability

Methods in this manuscript are available as a module in the Python package PyIOmica, https://doi.org/10.5281/zenodo.4542082 (Documentation: https://pyiomica.readthedocs.io/en/latest/ ). Datasets and codes used are available at https://doi.org/10.5281/zenodo.4542567.

## References

[1] Alon U (2006). An introduction to systems biology: design principles of biological circuits.

[2] Berk M, Ebbels T, Montana G (2011). A statistical framework for biomarker discovery in metabolomic time course data. Bioinformatics.

[3] Bar-Joseph Z, Gitter A, Simon I (2012). Studying and modelling dynamic biological processes using time-series gene expression data. Nat. Rev. Genet..

[4] Rose SMS-F (2019). A longitudinal big data approach for precision health. Nat. Med..

[5] Zhou W (2019). Longitudinal multi-omics of host-microbe dynamics in prediabetes. Nature.

[6] Ding J (2019). Integrating multi-omics longitudinal data to reconstruct networks underlying lung development. Am. J. Physiol. Lung Cell. Mol. Physiol..

[7] Piening BD (2018). Integrative personal omics profiles during periods of weight gain and loss. Cell Syst..

[8] Stanberry L (2013). Integrative analysis of longitudinal metabolomics data from a personal multi-omics profile. Metabolites.

[9] Chen R (2012). Personal omics profiling reveals dynamic molecular and medical phenotypes. Cell.

[10] Sherman BT (2009). Systematic and integrative analysis of large gene lists using David bioinformatics resources. Nat. Protoc..

[11] Giardine B (2005). Galaxy: a platform for interactive large-scale genome analysis. Genome Res..

[12] Reich M (2006). Genepattern 2.0. Nat. Genet..

[13] Mias GI (2016). Mathiomica: an integrative platform for dynamic omics. Sci. Rep..

[14] Domanskyi S, Piermarocchi C, Mias GI (2020). PyIOmica: longitudinal omics analysis and trend identification. Bioinformatics.

[15] Zhang J, Small M (2006). Complex network from pseudoperiodic time series: topology versus dynamics. Phys. Rev. Lett..

[16] Lacasa L, Luque B, Ballesteros F, Luque J, Nuno JC (2008). From time series to complex networks: the visibility graph. Proc. Natl. Acad. Sci..

[17] Luque B, Lacasa L, Ballesteros FJ, Robledo A (2011). Feigenbaum graphs: a complex network perspective of chaos. PLoS ONE.

[18] Donner RV (2011). Recurrence-based time series analysis by means of complex network methods. Int. J. Bifurc. Chaos.

[19] Campanharo AS, Sirer MI, Malmgren RD, Ramos FM, Amaral LAN (2011). Duality between time series and networks. PLoS ONE.

[20] Shimada Y, Ikeguchi T, Shigehara T (2012). From networks to time series. Phys. Rev. Lett..

[21] Xu X (2012). Single-cell exome sequencing reveals single-nucleotide mutation characteristics of a kidney tumor. Cell.

[22] Stephen M, Gu C, Yang H (2015). Visibility graph based time series analysis. PLoS ONE.

[23] Yang Y, Yang H (2008). Complex network-based time series analysis. Phys. A Stat. Mech. Appl..

[24] Zou Y, Donner RV, Marwan N, Donges JF, Kurths J (2019). Complex network approaches to nonlinear time series analysis. Phys. Rep..

[25] Bhaduri S, Ghosh D (2015). Electroencephalographic data analysis with visibility graph technique for quantitative assessment of brain dysfunction. Clin. EEG Neurosci..

[26] Elsner J, Jagger T, Fogarty E (2009). Visibility network of United States hurricanes. Geophys. Res. Lett..

[27] Telesca L, Lovallo M, Ramirez-Rojas A, Flores-Marquez L (2014). Relationship between the frequency magnitude distribution and the visibility graph in the synthetic seismicity generated by a simple stick-slip system with asperities. PLoS ONE.

[28] Telesca L, Lovallo M, Toth L (2014). Visibility graph analysis of 2002–2011 Pannonian seismicity. Phys. A Stat. Mech. Appl..

[29] Aguilar-San Juan B, Guzmán-Vargas L (2013). Earthquake magnitude time series: scaling behavior of visibility networks. Eur. Phys. J. B.

[30] Yang Y, Wang J, Yang H, Mang J (2009). Visibility graph approach to exchange rate series. Phys. A Stat. Mech. Appl..

[31] Suyal V, Prasad A, Singh HP (2014). Visibility-graph analysis of the solar wind velocity. Sol. Phys..

[32] Zou Y, Donner R, Marwan N, Small M, Kurths J (2014). Long-term changes in the north-south asymmetry of solar activity: a nonlinear dynamics characterization using visibility graphs. Nonlinear Process. Geophys..

[33] Shao Z-G (2010). Network analysis of human heartbeat dynamics. Appl. Phys. Lett..

[34] Ahmadlou M, Adeli H, Adeli A (2010). New diagnostic EEG markers of the Alzheimer’s disease using visibility graph. J. Neural Transm..

[35] Zhu G, Li Y, Wen PP, Wang S (2014). Analysis of alcoholic EEG signals based on horizontal visibility graph entropy. Brain Inform..

[36] Supriya S, Siuly S, Wang H, Cao J, Zhang Y (2016). Weighted visibility graph with complex network features in the detection of epilepsy. IEEE Access.

[37] Mira-Iglesias A, Conejero JA, Navarro-Pardo E (2016). Natural visibility graphs for diagnosing attention deficit hyperactivity disorder (ADHD). Electron. Notes Discrete Math..

[38] Wang L, Long X, Arends JB, Aarts RM (2017). EEG analysis of seizure patterns using visibility graphs for detection of generalized seizures. J. Neurosci. Methods.

[39] Sannino S, Stramaglia S, Lacasa L, Marinazzo D (2017). Visibility graphs for FMRI data: multiplex temporal graphs and their modulations across resting-state networks. Netw. Neurosci..

[40] Luque B, Lacasa L, Ballesteros F, Luque J (2009). Horizontal visibility graphs: exact results for random time series. Phys. Rev. E.

[41] Girvan M, Newman ME (2002). Community structure in social and biological networks. Proc. Natl. Acad. Sci..

[42] Clauset A, Newman ME, Moore C (2004). Finding community structure in very large networks. Phys. Rev. E.

[43] Newman ME (2006). Finding community structure in networks using the eigenvectors of matrices. Phys. Rev. E.

[44] Blondel VD, Guillaume J-L, Lambiotte R, Lefebvre E (2008). Fast unfolding of communities in large networks. J. Stat. Mech. Theory Exp..

[45] Li X (2017). Digital health: tracking physiomes and activity using wearable biosensors reveals useful health-related information. PLoS Biol..

[46] Liu, J., Liu, H., Huang, Z. & Tang, Q. Differ multivariate timeseries from each other based on a simple multiplex visibility graphs technique. In *2015 Sixth International Conference on Intelligent Control and Information Processing (ICICIP)* 289–295 (IEEE, 2015).

[47] Bezsudnov I, Snarskii A (2014). From the time series to the complex networks: the parametric natural visibility graph. Phys. A Stat. Mech. Appl..

[48] Hagberg, A. *et al.* in *Proceedings of the 7th Python in Science Conference (scipy2008)* (Dynamics, and Function Using NetworkX, Exploring Network Structure, 2008).

[49] Fredman ML, Tarjan RE (1987). Fibonacci heaps and their uses in improved network optimization algorithms. J. ACM.

[50] Mias GI (2021). Longitudinal saliva omics responses to immune perturbation: a case study. Sci. Rep..

[51] Mias GI, Zheng M (2020). The MathIOmica toolbox: general analysis utilities for dynamic omics datasets. Curr. Protoc. Bioinform..

[52] Sia J, Jonckheere E, Bogdan P (2019). Ollivier-Ricci curvature-based method to community detection in complex networks. Sci. Rep..

[53] Keogh, E., Chu, S., Hart, D. & Pazzani, M. Segmenting time series: a survey and novel approach. In *Data Mining in Time Series Databases* 1–21 (World Scientific, 2004).

[54] Gharghabi S (2019). Domain agnostic online semantic segmentation for multi-dimensional time series. Data Min. Knowl. Discov..

